# Acquired αSMA Expression in Pericytes Coincides with Aberrant Vascular Structure and Function in Pancreatic Ductal Adenocarcinoma

**DOI:** 10.3390/cancers14102448

**Published:** 2022-05-16

**Authors:** Vikneshwari Natarajan, Sangdeuk Ha, Alexander Delgado, Reed Jacobson, Lina Alhalhooly, Yongki Choi, Jiha Kim

**Affiliations:** 1 Department of Biological Science, North Dakota State University, Fargo, ND 58108, USA; v.natarajan@ndsu.edu (V.N.); sangdeuk.ha@ndsu.edu (S.H.); alexander.delgado@ndsu.edu (A.D.); jaco2784@umn.edu (R.J.); 2 Molecular and Cellular Biology Program, North Dakota State University, Fargo, ND 58108, USA; yongki.choi@ndsu.edu; 3 Department of Physics, North Dakota State University, Fargo, ND 58108, USA; lina.alhalhooly@ndus.edu

**Keywords:** pancreatic ductal adenocarcinoma (PDAC), pericytes, vascular leakiness, hypoxia, α-smooth muscle actin (αSMA), tumor microenvironment (TME), cancer exosomes

## Abstract

**Simple Summary:**

The pancreatic cancer field suffers from a lack of effective treatment options in part due to the severely compromised tumor vasculature that leads to hypoxia and an immunosuppressive environment. Our study aimed to identify and characterize pathological pericyte phenotypes that contribute to the defective tumor vasculature. We found that a large population of PDAC pericytes present ectopic α-smooth muscle actin (αSMA) expression, and this subtype was induced by cancer cell-derived exosomes. We also showed that the PDAC exosome-induced αSMA^+^ pericyte exhibits aberrant biomechanical properties and an acquired immunomodulatory phenotype. Considering a lack of efficient anti-angiogenic approaches in PDAC, identifying a unique molecular phenotype or the target molecule of a pathological pericyte will be an entry point toward a vascular normalization approach.

**Abstract:**

The subpopulations of tumor pericytes undergo pathological phenotype switching, affecting their normal function in upholding structural stability and cross-communication with other cells. In the case of pancreatic ductal adenocarcinoma (PDAC), a significant portion of blood vessels are covered by an α-smooth muscle actin (αSMA)-expressing pericyte, which is normally absent from capillary pericytes. The Desmin^low^αSMA^high^ phenotype was significantly correlated with intratumoral hypoxia and vascular leakiness. Using an in vitro co-culture system, we demonstrated that cancer cell-derived exosomes could induce ectopic αSMA expression in pericytes. Exosome-treated αSMA^+^ pericytes presented altered pericyte markers and an acquired immune-modulatory feature. αSMA^+^ pericytes were also linked to morphological and biomechanical changes in the pericyte. The PDAC exosome was sufficient to induce αSMA expression by normal pericytes of the healthy pancreas in vivo, and the vessels with αSMA^+^ pericytes were leaky. This study demonstrated that tumor pericyte heterogeneity could be dictated by cancer cells, and a subpopulation of these pericytes confers a pathological feature.

## 1. Introduction

Pancreatic ductal adenocarcinoma (PDAC) is the fourth leading cause of cancer death in both men and women in the US, with a five-year survival rate of 11% [1]. However, more than half of these patients are diagnosed after metastasis has occurred; for patients in this group, the five-year survival rate is decreased to 3% [1]. Reasons for this poor prognosis include the aggressive nature of the disease, lack of early detection methods, its complex and dense tumor microenvironment (TME) and lack of effective treatment options [2]. PDAC TME is composed of cancer-associated fibroblasts (CAFs), extracellular matrix (ECM), endothelial cells, perivascular cells or pericytes, immune cells and neurons [3]. Among the various components of the TME, tumor blood vessels orchestrate many tumor-specific features and aggressiveness. Tumor growth depends on a coordinated angiogenic response and vascular remodeling to meet the nutrient and oxygen demands posed by exponential growth [4,5,6,7,8]. Tumor vessels also serve as the main route for therapeutic drugs and immune cell trafficking [6,9,10,11,12,13], making it a double-edged sword for disease progression and targeting approaches. Tumor vessels are largely abnormal, leaky and lack proper pericyte coverage [9,14,15,16,17,18], contributing to elevated hypoxia, acidosis and interstitial fluid pressure [3,19], which promote cancer progression and metastasis [12,13,20,21]. In addition, nonfunctional tumor vessels pose a major obstacle to drug delivery and immune cell infiltration [12]. While tumor vasculature has a substantial number of pericytes, depending on the tumor type, the perivascular composition is heterogeneous and aberrant from the normal composition in healthy tissues. Several studies showed that pericyte coverage significantly correlates with vascular integrity/function and intratumoral hypoxia [22,23,24,25]. Depletion of specific types of pericytes augments metastatic dissemination in breast cancer [22], with pericytes as a gatekeeper in TME for multiple solid tumors [26]. In a preclinical mouse model of breast cancer, angiopoietin-2 (Ang2) neutralizing antibody treatment significantly improved the vascular structure, partly due to the recruitment of mature pericytes (Desmin^+^) and reduced hypoxia [23]. Several studies have reported pathological pericyte phenotypes and their different functions in breast cancer [24]. Ectopic expression of Klf4, Rgs-5, CD248 and mutant GT198 in tumor-associated pericytes can cause local and distant metastases in breast cancer and PNETs (pancreatic neuroendocrine tumors) [27,28,29,30]. Notably, recent studies reported ectopic expression of αSMA in pericytes of various disease models. αSMA is an actin isoform and contributes to cell-generated mechanical tension [31]. A recent report on pathological pericytes in ischemic retinopathy showed abnormal expression of αSMA induces pathological neovascular tuft formation [32]. Morikawa et al. showed that ectopic αSMA expression by tumor-associated pericytes correlates with PNET progression [16]. Ectopic expression of αSMA in capillary pericytes [33] is also known to confer a vascular smooth muscle cell (vSMC)-like phenotype [34,35]. Although abnormal pericytes confer pathological features of the vessels, a recent study showed that PDAC patients with more blood vessels have better overall survival [36]. These reports indicate that an anti-angiogenic approach to deplete/prune tumor vessels will likely yield more aggressive tumors, especially in PDAC [22,37,38]. Therefore, understanding the nature of the pathological phenotype of the pericyte is critical before investigating a novel way to design an anti-vascular approach. Pericytes are known for their plasticity, versatility and expression of heterogeneous markers [14,39], and specific pericyte phenotypes are largely influenced by their local environment [24,40]. Structurally, pericytes share a basement membrane with endothelial cells, allowing intimate reciprocal communication [14]. Adhesion molecules are important for this anatomical position and signaling [41]. Pericytes are also important for cellular trafficking, especially immune cells [42,43]. While physical contact with endothelial cells is required for pericytes to mature during angiogenesis [7], exactly what triggers abnormal pericyte phenotypes during pathological angiogenesis is unclear. Several factors have been suggested to cause the pericyte phenotype shift, including TGF-β [7], PDGF-BB [32] and Ang2 [22]. However, the functional consequence and molecular signature of altered pericytes remain unknown.

In this study, we sought to delineate the unique perivascular phenotype of PDAC tumors using various mouse models and human tissue microarray (TMA). Robust imaging analysis demonstrated that tumor-associated pericytes express ectopic αSMA, which is significantly correlated with hypoxia and vascular leakiness. Through an in vitro vascular cell culture, we clearly showed that the pericyte phenotype is strongly influenced by cancer cell-derived exosomes. We further demonstrated that αSMA^high^ pericytes present morphological and biomechanical differences on PDAC-Exo exposure. In addition, these pericytes acquired immunomodulatory gene expression, suggesting a potential contribution of tumor-associated pericytes to immunosuppressive TME of PDAC.

## 2. Material and Methods

### 2.1. Animal Studies

The disease progression and genotyping for KPC mice were previously described [44]. To establish KPC mice, Kras-LSL-G12D (JAX 008180) and Pdx-1-Cre (JAX, 014647) mice were purchased from Jackson Laboratory and bred in the NDSU animal facility. The p53-LSL-R172H line was established through cryo-recovery using cryo-embryos from the Frederick National Laboratory for Cancer Research (01XAF, B6.129S4-Trp53 < tm2Tyj) [45]. Three lines were bred at the NDSU animal facility to generate KPC mice. To that end, C57BL/6J (000664) was purchased from Jackson Laboratory, while NOD-SCID IL2Rgamma ^null^ (NSG) mice were provided by the NDSU Small Animal Core Facility. NSG immunocompromised mice between four and six weeks of age were housed in individually ventilated cages on a 12-h light:12-h dark cycle. Under general anesthesia, 1 × 10^6^ PANC-1 cells suspended in 20 ul of PBS were injected into the tail of the pancreas using a 25-gauge syringe. After 28 days, the mice were euthanized, and pancreatic tumors were harvested. For the exosome injection experiment, NSG mice were injected with HPNE or PANC-1 exosomes (10^10^ exosomes per injection) intraperitoneally in 100 μL of PBS every other day for three weeks. WT C57BL/6J mice were injected with KPC exosomes as described above. KTC tumor tissues were provided by Dr. Kalluri. All animal experiments were reviewed and approved by the Institute of Animal Care and Use Committee of the North Dakota State University.

### 2.2. Vascular Leakage

Mice were injected in the retro-orbital venous plexus with 100 μL of 10 mg/mL FITC-Dextran (2,000,000 MW, Sigma) 5 min before euthanasia. Pancreatic tumors and normal pancreases were harvested and fixed in 10% neutral buffered formalin. Paraffin-embedded sections were immunostained for CD31 to visualize the vessels. CD31 and FITC-Dextran were then visualized directly by fluorescent microscopy under red and green fluorescent filters. Fluorescent images were taken under 20× magnification, with at least three tumors/experimental group. Images were quantified using NIH ImageJ analysis software. The proportion of leaked FITC-Dextran was calculated by subtracting the intravascular FITC-Dextran from the total FITC-Dextran present on the individual image.

Total FITC-Dextran (%)—FITC-Dextran within vessels (%) = leaked FITC-dextran.

### 2.3. Immunofluorescence Staining

Pancreatic tumors and normal pancreas tissues were harvested from mice, fixed in 10% neutral buffered formalin, embedded in paraffin and sectioned into 5 µm-thick sections. For antigen retrieval, these sections were deparaffinized, rehydrated and heated in TE buffer (pH 9.0) at 95° for 30 min (EZ Retriever microwave, BioGeneX; Fremont, CA, USA. For αSMA and Desmin staining, sections were blocked with M.O.M. mouse IgG blocking reagent (Vector Laboratories; Burlingame, CA, USA) for 1 h. Sections were incubated with mouse anti-αSMA antibody (1:400, Sigma; Saint Louis, MO, USA and mouse anti-Desmin antibody (1:50, Invitrogen; Waltham, MA, USA) overnight at 4 °C, followed by incubation with biotin-conjugated anti-mouse IgG (Vector Laboratories) for 10 min. For all other staining, sections were heated for antigen retrieval in 10 mM citrate buffer at pH 6.0 at 95° for 15 min before blocking for 1 h with 4% cold water fish gelatin (CWFG) in phosphate-buffered saline w/Tween 20 (PBST) at room temperature. Following blocking, sections were incubated with rat anti-CD31 (1:100, Dianova; Hamburg, Germany), α-smooth muscle-FITC conjugated (1:500, Sigma), rabbit anti-CA9 (1:000, Invitrogen), rabbit anti-PDGFRB (1:100, Invitrogen) or rat anti-CK8 (1:50, DSHB; Iowa City, IA, USA) at 4 °C overnight. For fluorescent visualization of these antibodies, the secondary antibody used was: Streptavidin, Alexa Flour™546 conjugated (1:250, Invitrogen), Cy5™ anti-rat IgG (1:250, Invitrogen) or Alexa Flour™546 anti-rabbit IgG (1:250, Invitrogen), which was incubated for 1 h at room temperature. Slides were mounted using Fluoroshield™ mounting media with DAPI to label nuclei. Antibody positivity was analyzed in 5–8 visual fields at 20× magnification for all staining.

FFPE human TMA blocks were deparaffinized and rehydrated before antigen retrieval in heated TE buffer (pH 9.0) for 30 min. Each TMA section was sequentially stained for different primary antibodies. Each round of staining included a blocking step with 4% CWFG in PBST followed by primary antibody incubation and a corresponding secondary HRP-conjugated polymer (SuperPicture, Invitrogen). Each HRP-conjugated polymer mediated the covalent binding of a different fluorophore (FITC, Cy3 and Cy5) using Tyramide Signal Amplification (PerkinElmer). This covalent reaction was followed by additional antigen retrieval in heated citric acid buffer (pH 6.0) for 15 min to remove bound antibodies before the next step in the sequence, which allows for the use of multiple primary antibodies with the same species origin. After sequential reactions, sections were counterstained with DAPI (Life Technologies; Carlsbad, CA, USA) and mounted with VECTASHIELD Antifade Mounting Medium (Vector Laboratories). The primary antibodies used for this protocol were: rabbit anti-CA9 (1:1000, Invitrogen), rabbit anti-CD31 (1:4000, Bethyl Laboratories; Montgomery, TX, USA), anti-αSMA (1:2000, Sigma) and anti-Desmin (1:600, Abcam; Waltham, MA, USA).

### 2.4. Colocalization Analysis of Pericyte Coverage

Images of four-color immunolabeled sections from experimental groups were subjected to colocalization analysis. In this process, overlapping areas between CD31 (endothelial cells) and αSMA (pericytes) or CD31 (endothelial cells) and Desmin (pericytes) were measured in ImageJ software using colocalization application. Overlapping of pericyte marker-expressing cells with CD31^+^ cells indicates physical contact between pericytes and endothelial cells. Large vessels associated with αSMA-expressing smooth muscle cells that wrap around the vessels horizontally were excluded from the imaging and quantification if they remained on the images.

### 2.5. Human Patient Samples

Human PDAC tissue microarrays were purchased from Biomax.us (T 144a, T142c and PA482). PDAC tissues and normal pancreatic tissues were subjected to immunofluorescent staining using the TSA system described above.

### 2.6. Ang2 ELISA

Blood samples were collected in EDTA-containing tubes and spun down for 20 min at 2000 g and 4 °C. Plasma was collected and stored at −80 °C until use. Serial dilutions of plasma samples were tested for the optimal protein concentration. Plasma was diluted in the sample diluent buffer provided with the Angiopoietin-2 Mouse ELISA Kit (Abcam, ab171335), and the ELISA was performed following the manufacturer’s directions.

### 2.7. Cell Culture

*Cancer cells.* PANC-1 was obtained from ATCC and cultured in high-glucose DMEM supplemented with 10% FBS and 1% penicillin-streptomycin. KPC 689 cells were kindly provided by Dr. Kalluri (MD Anderson Cancer Center, Houston, TX, USA) and cultured in RPMI supplemented with 10% FBS, 10 mM HEPES and 1% penicillin-streptomycin. HPNE was obtained from ATCC and cultured in high-glucose DMEM supplemented with 10% FBS, 1% penicillin-streptomycin and 0.75 ug/mL puromycin.

*Primary Cells.* Human pericytes from placenta (C-12980 hPC-PL, PromoCell; Heidelberg, Germany ) were cultured in pericyte growth medium containing PromoCell SupplementMix (PromoCell; C-28041). HUVECs (PromoCell, C-12200) were cultured in endothelial cell basal Medium 2 containing PromoCell SupplementPack (PromoCell C-22110). Primary cells were only used up to passage six for the experiments.

### 2.8. Quantitative Real-Time PCR Analyses

Cells were lysed using TRIzol® Reagent (Invitrogen), and the total RNA was extracted using a Direct-zol RNA MiniPrep Kit according to the manufacturer’s instructions. Each RNA sample was prepared in triplicate, and qPCR reactions were run with technical triplicates. We used 1 μg of total RNA to synthesize cDNA using the High-Capacity cDNA Reverse Transcription Kit (Applied Biosystems; Waltham, MA, USA). Gene expression was determined using the QuantStudio 3 Real-Time PCR System and Power SYBR Green PCR Master Mix (Applied Biosystems). Measurements were standardized to the housing-keeping gene (beta-2-microglobulin, B2M) or PECAM1 for endothelial cells. Primer sequence information is listed in Table S1.

### 2.9. Exosome Isolation and Quantification

PANC-1, HPNE and KPC cells were cultured in their respective media supplemented with 10% exosome-depleted FBS (Invitrogen) for exosome isolation. Once cells were grown sub-confluent, the medium was changed to contain exosome-free serum and cultured for an additional 48 h. Culture supernatant was subjected to sequential centrifugation steps at 800 g for 5 min and 2000 g for 10 min, then filtered with 0.2 mm filters. The filtrate was subjected to ultracentrifugation at 28,000 rpm using an SW 28 Ti swinging bucket overnight to collect exosomes. The supernatant was aspirated, and the pellet was resuspended in PBS. The purified exosomes were then analyzed for quantity and size using NanoSight and used for experimental procedures.

### 2.10. Visualization of Exosomes in Cells

Exosomes isolated from HPNE and PANC-1 cells were resuspended in 1 mL PBS, and 2 μL (1:500) DiO’; DiOC18(3) dye was added. The thoroughly mixed solution was incubated at 37 °C for 5 min and then at 4 °C for 10 min. The mixture was ultracentrifuged for 2 h and the pellets were resuspended in PBS. Labeled exosomes were added to pericytes cultured on glass coverslips for 2 h. The cells were visualized for green fluorescence (exosomes) and bright-field (cell morphology) using a ZOE Fluorescent Cell Imager (Bio-Rad; Hercules, CA, USA).

### 2.11. Exosome Treatment for Cultured Cells

For the exosome treatment experiment, hPCs or hECs (HUVECs) were cultured in a six-well plate coated with attachment factor protein (GibcoTM). For each well, 150,000 cells were seeded except when hPC was co-cultured with either HUVEC or KPC-689 cancer cells, in which case, 75,000 cells of each line were seeded together. For each experimental group, wells were triplicated. Once cells reached 70% confluency, the medium was replaced with basal medium for starvation. After 6 h, cells were treated with 2 × 10^9^ exosomes per well for 24 h, then harvested for total RNA extraction. For immunocytochemistry of pericytes treated with exosomes, cells were washed with cold PBS and incubated with 4% PFA at room temperature for 15 min. The cells were then washed with PBS, incubated with 0.05% Triton X-100 for 10 min, washed with PBS and stained with mouse anti-α-smooth muscle actin—FITC antibody (1:500, Sigma) and Alexa Fluor™ 594 Phalloidin. The coverslips were then mounted onto glass slides with mounting media containing DAPI. Images were taken with a Zeiss Axio Scope A1 upright compound microscope. The maximum Feret’s diameter, roundness and aspect ratio were measured by ImageJ software. Individual images captured 5–10 cells, and five images per well were used for quantification.

### 2.12. Atomic Force Microscopy

The cell imaging and spectroscopy measurements were conducted using a commercial AFM (NT-MDT NTEGRA) with an optical viewing system and V-shaped silicon nitride AFM probes as previously described [46]. Briefly, we used a cantilever with a spring constant of 0.08 N/m and tip face angle of 35° (Nanoworld; Neuchâtel, Switzerland). The cantilever spring was calibrated by the thermal noise fluctuation methods [47] and the deflection sensitivity of each tip was calibrated by force-distance curve measurements on the bare glass area of the petri dish. At least seven cells were randomly selected for all imaging and other force measurements. The scanning resolution was 256 × 256 pixels with a scan rate of 0.1–0.5 Hz, depending on the scanning areas of irregular cell size. The acquired images were flattened, if required, to eliminate the background noise and tilt from the surface using all unmasked portions of scan lines to calculate the individual least-squares fit polynomials for each line.

### 2.13. Stiffness and Adhesion Measurements

The relative cell stiffness (Young’s modulus *E*) and cell surface adhesion were extracted from force-distance (FD) curves as described previously [46]. The FD curves were obtained on the central cytoplasmic region of the cell surface. The approaching and retracting rates of the probe were 1 µm/s for all measurements. To prevent cell damage and eliminate potential substrate effects, force measurements were performed with a shallow indentation depth of cells (400 nm). Young’s modulus was determined by fitting the FD curves to the Hertz model [46,48,49,50]. The FD curve measurements typically involve non-specific adhesion between the macromolecules on the cell surface and the AFM probe tip. During tip retraction from the cell surface, the detachment force (adhesion force) required to separate the tip from the macromolecules on the cell surface was determined from a rupture event in a sawtooth-like pattern. The adhesion force was determined as the difference between force values at the zero-force line of the FD curve and at the negative minimum of the FD curve.

### 2.14. Nanoparticle Tracking Analysis

The size distribution and concentration of exosomes were determined by nanoparticle tracking analysis (NTA) using the NanoSight NS300 system (Malvern Panalytical Ltd., Malvern, UK). The exosome samples were diluted 1000-fold in PBS for NTA measurements. The samples were infused with the syringe pump at a constant speed of 20 into the microfluidic flow cell equipped with a 53- nm laser and a high-sensitivity scientific CMOS camera. At least three videos per sample were recorded with the camera level of 11–13 for 30 s at 25 °C. All data were analyzed using NTA software (version 3.4; Malvern Panalytical Ltd., Malvern, UK) with a detection threshold of 5.

### 2.15. Statistical Analysis

n corresponds to the number of animals; N corresponds to the number of independent experiments performed for qRT-PCR. In correlation plots, individual values from each image were plotted. From each tumor (animal), 5–10 images were used for the analysis. For comparison between two groups with one grouping variable, Student’s unpaired two-tailed *t*-test was used. To compare multiple groups with one grouping variable, one-way ANOVA with multiple comparisons and Tukey’s correction was performed. To compare multiple groups with two grouping variables, two-way ANOVA with Šídák’s multiple comparisons test was used. Statistical analysis was performed using Prism 9 (version 9.0.1; GraphPad software, San Diego, CA, USA). Data were averaged and expressed with means ± SEM and values of *p* < 0.05 were considered statistically significant.

## 3. Results

### 3.1. PDAC-Associated Pericyte Exhibits Ectopic αSMA Expression

**Figure 1:**
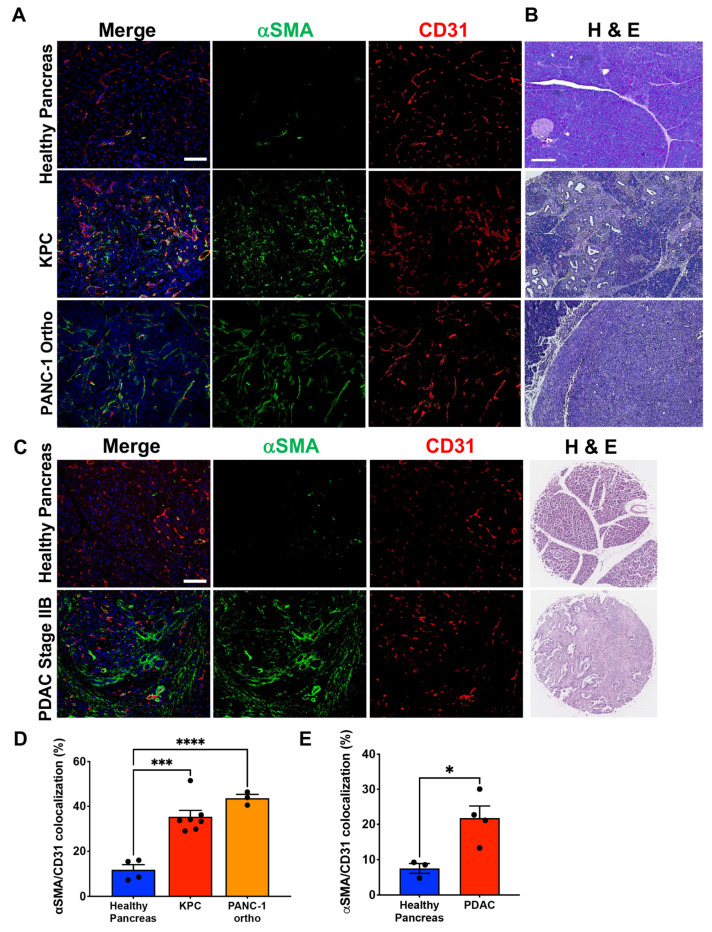
PDAC-associated pericytes exhibit aberrant molecular phenotype. (**A**) Representative images of FFPE sections of KPC and PANC-1 tumors, and healthy pancreas tissues immunolabeled for CD31 and αSMA. Sections are counterstained with DAPI (blue) to visualize nuclei. (**B**) Representative images of H&E stained KPC and PANC-1 tumors, and healthy pancreas tissues. Scale bar: 200 μm. (**C**) Representative images of FFPE tissue microarray sections from normal human pancreas and PDAC tumors immunolabeled for CD31 and αSMA or H&E-stained. Scale bar: 100 μm. (**D**) Quantification of percentage of CD31^+^ vessels associated with αSMA^+^ pericytes (WT *n* = 4, KPC *n* = 7 and PANC-1 ortho tumors *n* = 3). One-way ANOVA with Dunnett’s multiple comparisons was used to determine statistical significance. (**E**) Quantification of percentage of CD31^+^ vessels associated with αSMA^+^ pericytes (healthy pancreas *n* = 3, PDAC *n* = 4). Unpaired two-tailed *t*-test was used to determine statistical significance. Scale bar: 100 μm; apply to all images. Unless otherwise stated, the data are represented as the mean ± SEM. * *p* < 0.05, ** *p* < 0.001, *** *p* < 0.0001.

Pericyte coverage is critical for vascular structure and function, and its significant heterogeneity has been studied in several solid tumors. However, comprehensive perivascular phenotyping has not been investigated in PDAC despite the severely compromised vascular functionality. Therefore, we investigated perivascular landscaping in PDAC tumors using various mouse models for PDAC and TMAs constructed using resected tumors of PDAC patients. Tumor tissues from two GEMMs, KPC and KTC, and three orthotopic models, PANC-1, MiaPaCa-2 and HPAF-II, were stained for CD31(endothelium), αSMA and Desmin (Figure 1 and Figure S1A,B,D,E). For KPC and PNAC-1 tumors, tissues were also stained for CK8 to identify cancer cells (Figure S2). To exclude non-perivascular mesenchymal cells such as tumor-associated fibroblasts that express the same markers, we only quantified perivascular cells physically attached to the endothelium using a co-localization function of ImageJ software. One of the most prominent changes, when compared to the normal pancreas tissues, was the ectopic expression of αSMA in tumor-associated pericytes across all PDAC tumor tissues. Up to ~73% of vessels were associated with αSMA^+^ pericytes in murine models of PDAC, which is 13 times higher than that of the normal pancreas (Figure 1A,B,D and Figure S1A,B). Similarly, 22% of vessels were covered by αSMA^+^ pericytes in human PDAC tumors, which is about 2.5 times higher than normal pancreatic vessels (Figure 1C,E). As previously reported [16], a significant portion of pancreatic neuroendocrine tumor (PNET) vessels were also covered by αSMA^+^ pericytes (Figure S1C,F). αSMA immunostaining on KPC tumors at different stages shows the progressive appearance of αSMA^+^ pericytes, with strong perivascular localization on capillaries at 14 weeks (Figure S3A,B). Notably, αSMA ^+^ pericytes were present on a small fraction of vessels associated with PanIN lesions, whereas most, if not all, vessels within adjacent healthy tissue were not associated with αSMA^+^ pericytes (Figure S3A,C). A similar phenotypic shift across different PDAC models indicates that these cells might be undergoing molecular phenotype changes in response to stimulation in the TME. This result suggests that ectopic αSMA expression defines the pathological phenotype of PDAC-associated pericytes.

### 3.2. Perivascular Phenotype Shift Is Correlated with Vascular Integrity/Function

**Figure 2:**
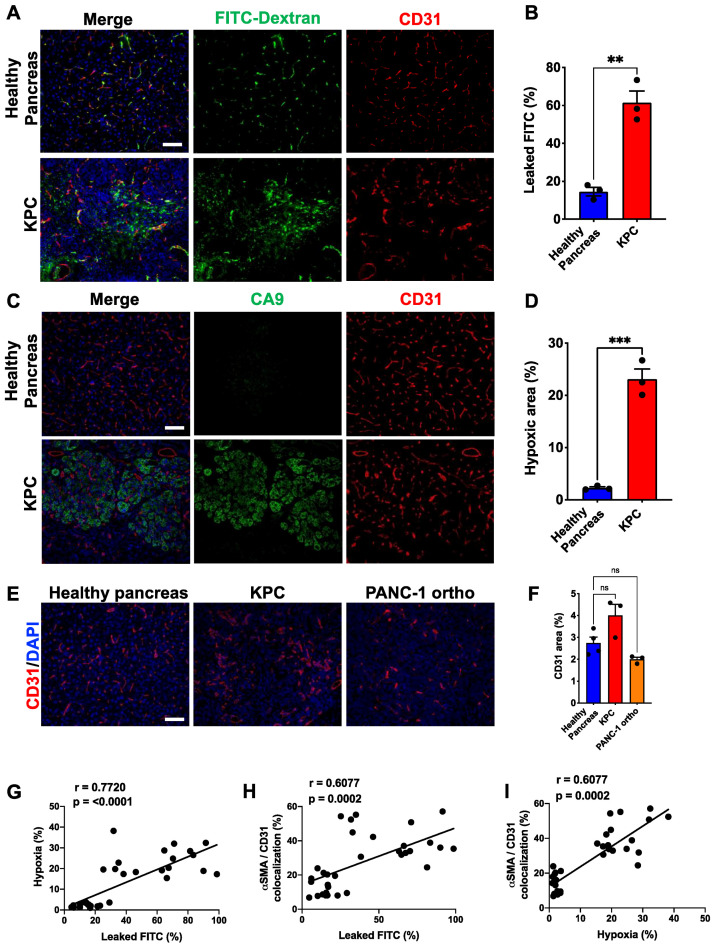
αSMA^+^ pericytes’ coverage is significantly correlated with vessel leakiness and hypoxia. (**A**) Representative images of FFPE sections of KPC tumors and healthy pancreas immunolabeled for CD31. Tissue sections were also visualized using perfused 2000-kDa FITC-dextran in the indicated groups. Sections are counterstained with DAPI (blue) to visualize nuclei. (**B**) Quantification of percentage of FITC-dextran leaked to the tissue (*n* = 3, all groups). (**C**) Representative images of FFPE sections of KPC tumors and healthy pancreas. Sections were immunolabeled for carbonic anhydrase 9 (CA9; hypoxia) and CD31. (**D**) Quantification of percentage of hypoxic area (CA9^+^ area per image) (*n* = 3, all groups). (**E**) Representative images of KPC and PANC-1 tumors, and healthy pancreas tissues stained for CD31. (**F**) Quantification of MVD (WT *n* = 4; KPC and PANC-1 ortho-tumors *n* = 3). One-way ANOVA with Dunnett’s multiple comparisons was used to determine statistical significance. (**G**) Correlation of FITC-dextran leakage and hypoxic area (*n* = 6). (**H**) Correlation of FITC-dextran leakage and percentage of αSMA^+^ pericyte-covered vessels (*n* = 6). (**I**) Correlation of hypoxic area and αSMA^+^ pericyte-covered vessels (*n* = 6). Pearson’s r correlation coefficient and significance levels are presented. Scale bar: 100 μm; apply to all images. Unless otherwise stated, the data are represented as the mean ± SEM, and an unpaired two-tailed *t*-test was used to determine statistical significance. ** *p* < 0.01, *** *p* < 0.001, ns = not significant.

Pericytes are a critical component of intact vasculature for intercellular communication and structure. Therefore, we next characterized the structural and functional competence of PDAC tumor vessels using KPC and PANC-1 orthotopic tumor models. We injected 2000 kDa FITC-Dextran retro-orbitally into 20-week-old tumor-bearing KPC mice and age-matched healthy WT mice and let FITC-Dextran circulate for 5 min before tumors were harvested. As 2000 K FITC-Dextran cannot extravasate intact blood vessels, the presence of FITC-Dextran in tumor tissue outside of the blood vessels indicates leaky vasculature. Of the entire FITC signal detected per each image of the KPC tumor, more than 60% was outside the vessels, whereas most of the FITC signal was detected within the vessels in case of normal pancreatic tissue, indicating healthy intact vessels (Figure 2A,B). Leaky vasculature leads to insufficient oxygen delivery, thus inducing a hypoxic tumor environment. So, we stained healthy pancreas and KPC tumor tissues with carbonic anhydrase 9 (CA9), indicative of hypoxia. While normal pancreatic tissues showed no detectable CA9 signal, ~25% of KPC tumor tissues were hypoxic (Figure 2C,D). A similar result in the hypoxic area was also observed in PANC-1 orthotopic tumors (Figure S4A,B). In human PDAC, both the αSMA^+^ pericyte coverage and CA9^+^ hypoxic area were significantly higher in stage-III than stage-I tumors, suggesting progressive deterioration of tumor vasculature over time (Figure S4C). Although the PDAC tumors presented significantly higher hypoxia, the overall microvascular density (MVD) showed no differences between the healthy pancreas and PDAC tumors, indicating that hypoxia is due to the quality of vessels rather than their quantity (Figure 2E,F). Overall, vascular leakiness, measured by FITC-Dextran, was significantly correlated with tumor hypoxia and αSMA^+^ pericyte coverage (Figure 2G,H). The αSMA^+^ pericyte coverage was also correlated with tumor hypoxia (Figure 2I). This result strongly supports our hypothesis that ectopic αSMA expression confers a pathological phenotype to PDAC-associated pericytes.

### 3.3. Desmin^low^/αSMA^high^ Phenotype Is Correlated with Vessel Leakiness and Hypoxia

**Figure 3:**
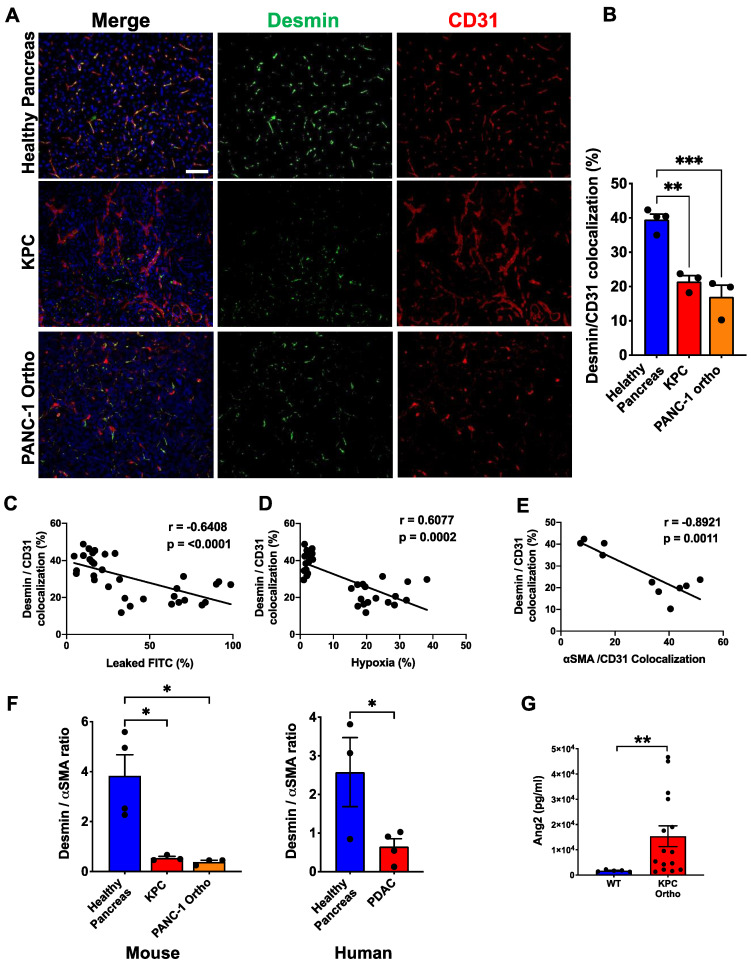
Desmin^low^/αSMA^high^ phenotype is correlated with vessel leakiness and hypoxia. (**A**) Representative images of FFPE sections of KPC and PANC-1 tumors, and healthy pancreas immunolabeled for Desmin and CD31. Sections are counterstained with DAPI (blue) to visualize nuclei. (**B**) Quantification of percentage of CD31^+^ vessels associated with Desmin^+^ pericytes. (WT *n* = 4; KPC and PANC-1 ortho-tumors *n* = 3). One-way ANOVA with Dunnett’s multiple comparisons was used to determine statistical significance. (**C**) Correlation of FITC-dextran leakage and Desmin^+^ pericyte-covered vessels (*n* = 6). (**D**) Correlation of hypoxic area and Desmin^+^ pericyte-covered vessels (*n* = 6). (**E**) Correlation of Desmin^+^ pericyte and αSMA^+^ pericyte-covered vessels (*n* = 10). Pearson’s r correlation coefficient and significance levels are presented. (**F**) The ratio of Desmin^+^ pericyte and αSMA^+^ pericyte per each tissue section was compared between the healthy pancreas and PDAC tumors for both murine models and humans. (**G**) Plasma Ang2 level was compared between KPC tumor-bearing mice and healthy mice using the Ang2 ELISA kit. Unpaired two-tailed *t*-test was used to determine statistical significance. Scale bar: 100 μm; apply to all images. Unless otherwise stated, the data are represented as the mean ± SEM. * *p* < 0.05, ** *p* < 0.01, *** *p* < 0.001.

Our previous breast cancer study showed that the ratio of different markers is indicative of pericyte characteristics and functionality. In particular, a high Desmin^+^/PDGFRβ^+^ ratio was correlated with better patient outcomes for those who received specific neoadjuvant therapy [23]. We also investigated the pericyte phenotype in which the ratio between αSMA and Desmin was quantified. In the case of PDAC tumors, the percentage of vessels covered by Desmin^+^ pericytes was reduced by 50% compared to that of the healthy pancreas (Figure 3A,B). Overall, the Desmin^+^ pericyte coverage was inversely correlated with vessel leakiness, hypoxia and the αSMA^+^ pericyte coverage (Figure 3C,D). A decrease in Desmin^+^ pericytes and an increase in αSMA^+^ pericytes resulted in an up to 10 times lower Desmin^+^/αSMA^+^ ratio in murine PDAC tumor models and four times lower in human PDAC tumors (Figure 3F). These data suggest that a perivascular phenotype shift from Desmin^high^/αSMA^low^ to Desmin^low^/αSMA^high^ contributes to the overall quality of the tumor vasculature, leading to significantly leaky vessels associated with the hypoxic tumor microenvironment.

The underlying mechanism that controls the dynamic switching of the pericyte molecular signature is largely unknown. We have previously shown that the Ang2 level correlates with the pericyte phenotype, in which lower Ang2 results in a more mature and stable pericyte phenotype (Desmin^high^/PDGFRβ^low^). When we measured the plasma Ang2 level by ELISA, orthotopic KPC tumor-bearing mice showed increased circulating Ang2 compared to healthy mice (Figure 3G). This finding partly explains the Desmin^low^/αSMA^high^ pericyte phenotype in PDAC. However, while a high Ang2 level might contribute to the Desmin^low^ phenotype, the cause of ectopic αSMA expression needs further elucidation.

### 3.4. PDAC Cell-Derived Exosomes Manipulate the Pericyte Phenotype and Influence the Quality of the Tumor-Associated Vasculature

To understand the underlying mechanism of ectopic αSMA expression in PDAC-associated pericytes, we established an endothelial cell (hEC; HUVEC)/pericyte co-culture (hPC; hPC-PL) system and analyzed the gene expression profile of a pericyte on exposure to environmental factors. We exposed pericytes to various conditions such as physical contact with cancer cells or hECs, hypoxia, changes in media composition, cancer cell-conditioned medium or cancer cell-derived exosomes. The gene expression profile and basal gene expression level of both hEC and hPC were established before exposure to external factors (Figure S5A). Of note, PECAM1 was exclusively expressed by hEC while all pericyte markers, CSPG4, PDGFRB, DES and ACTA2, were only within a detectable range in hPC. This finding confirms the cellular identity and rules out the possibility of these markers being expressed by endothelial cells in the co-culture system. 

**Figure 4:**
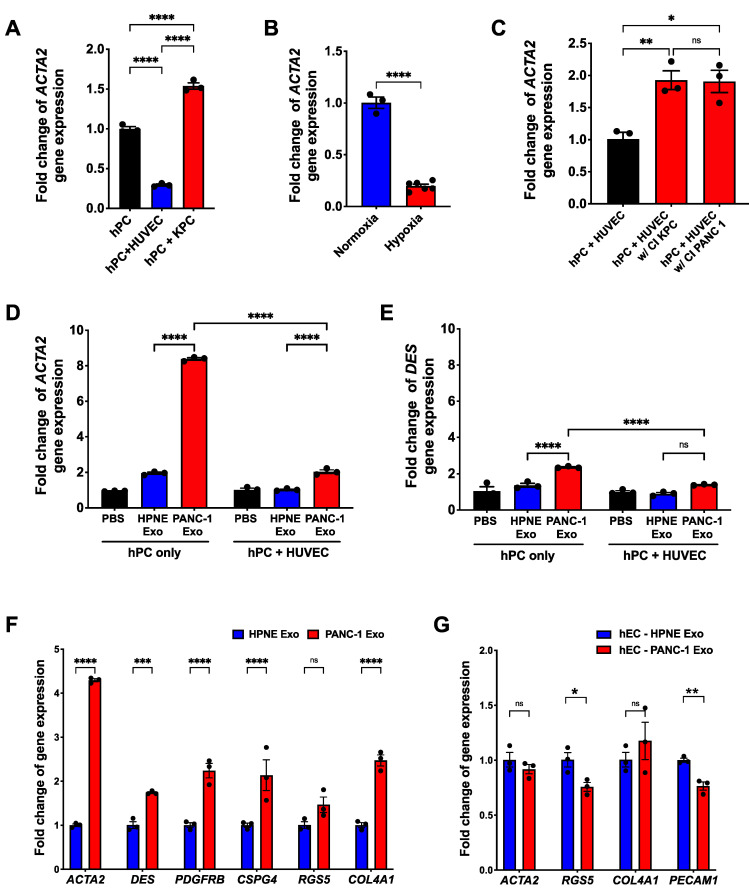
Pericytes’ phenotype is influenced by pancreatic cancer cells. (**A**–**C**) Quantification of *ACTA2* expression when pericytes are cultured in various conditions. (**A**) hPC was culture alone, with hEC (HUVEC), or KPC 689 cancer cells. (**B**) hPC was cultured in either a normoxic or hypoxic condition. (**C**) hPC and hEC were co-cultured at the bottom of a culture insert, and cancer cells (KPC or PANC-1) were cultured on the culture insert. (**D**,**E**) Either hPC alone or a hPC + hEC co-culture was treated with PBS, HPNE Exo or PANC-1 Exo. *ACTA2* (**D**) and *DES* (**E**) expression levels were measured by qRT-PCR after exosome treatment. (**F**) Quantification of pericyte marker gene expression of hPC treated with HPNE Exo or PANC-1 Exo. (**G**) Quantification of endothelial cell gene expression of hEC treated with HPNE Exo or PANC-1 Exo. Endothelial gene expression was normalized by *PECAM1.* One-way ANOVA with Tukey’s multiple comparisons test was used for (**A**,**C**–**E**), unpaired two-tailed *t*-test was used for (**B**) and two-way ANOVA with Šídák’s multiple comparisons test was used for (**F**,**G**) to determine statistical significance. *N* = 3 for all experiments and the data are represented as the mean ± SEM. * *p* < 0.05, ** *p* < 0.01, *** *p* < 0.00, **** *p* < 0.000, ns = not significant.

First, we co-cultured hPC with either hECs or murine PDAC cells (KPC689) and measured the relative expression levels of αSMA and Desmin (Figure 4A). While there was no significant difference in Desmin expression, co-culture with KPC 689 stimulated αSMA expression. hPC co-cultured with hEC showed significantly lower and barely detectable αSMA expression. PDAC TME is extremely hypoxic, and KPC tumor-bearing mice exhibited a significantly high level of Ang2, which is also known to be induced by hypoxia. Thus, we compared the αSMA expression when pericytes were cultured in hypoxic or normoxic conditions (Figure 4B). Interestingly, αSMA expression was significantly reduced by a hypoxic environment. While hypoxia is a potent environmental factor, influencing several aspects of cancer cells and TME, this result might indicate that the pericyte phenotype conversion requires a more direct molecular signal from either cancer cells or other stromal cells. To measure the overall effects of pancreatic cancer cells on the vasculature without physical contact, hPC and hEC were co-cultured with a culture insert where pancreatic cancer cells were grown as a monolayer on the surface of the membrane (Figure 4C). Both cancer cell lines, KPC689 and PANC-1, could induce αSMA expression in pericytes even without physical contact, suggesting the effect is via extracellular secretion from cancer cells. 

Considering this result, we next investigated the effect of PDAC cancer cell-derived exosomes on αSMA expression in pericytes. Exosomes are known to be representative of their cell of origin and act as mediators of intercellular communication in health and disease [51,52]. Exosomes were isolated from PANC-1 and KPC 689, and HPNE cells were used as a control via a standard ultracentrifugation procedure. The quantity and size distribution of exosomes were measured by Nanosight (Figure S5B). Exosome uptake by pericytes was confirmed by fluorescently labeled exosomes inside the pericytes (Figure S5C). Either hPC alone or a hPC + hEC co-culture were starved in basal media for 6 h and treated with exosomes for 24 h. While PBS and HPNE exosomes (HPNE Exo) showed minimal to no effects on the gene expression pattern, PANC-1 exosomes (PANC-1 Exo) induced αSMA expression that was significantly greater than for control groups (Figure 4D and Figure S5D). 

In accordance with the previous observation (Figure 4A), induction of αSMA expression by PANC-1 Exo was suppressed in the presence of endothelial cells (hPC + hEC co-culture, Figure 4D), suggesting that physical contact with endothelial cells stabilizes the pericyte phenotype. The same phenomenon was observed when the hPC + hEC co-culture was treated with KPC 689 exosomes (KPC Exo) (Figure S5E,F). While the PDAC exosome induced a slight increase of Desmin expression (Figure 4E), significantly higher αSMA expression skewed the overall phenotype to Desmin^low^/αSMA^high^, resembling the PDAC pericyte phenotype. Of note, PANC-1 Exo treatment led to mild induction of the other pericyte markers’ expression except for Rgs-5 (Figure 4F). Interestingly, Col4a1 expression was noticeably increased by PANC-1 Exo compared to the control (Figure 4F and Figure S5D). To assess the effect of PDAC exosomes on the endothelial cell phenotype, hEC was treated with either HPNE or PANC-1 Exo. However, only mild changes in PECAM1 and RGS5 were observed (Figure 4G), suggesting pericytes are more susceptible to environmental changes than endothelial cells.

### 3.5. Biomechanical Properties of Pericyte Are Affected by PDAC-Exo Treatment

Pericytes are a critical component of intact vasculature for intercellular communication and structure. αSMA is a cytoskeletal protein, and considering the significant increase of αSMA expression on exposure to PDAC exosomes, we investigated the biomechanical properties of pericytes with an altered phenotype. First, we measured the atomic force microscopy (AFM)-based stiffness of pericytes treated with HPNE Exo or PANC-1 Exo according to a previously established protocol [46]. Pericytes were cultured sparsely in 35-mm glass-bottom tissue culture dishes, and AFM measurements were obtained. Changes in cell stiffness (Young’s modulus *E*) were measured over the nuclear region of the cells in the medium. The cells were imaged using a silicon nitride AFM tip and indented by the cantilever to obtain the force-distance (FD) curves. 

**Figure 5:**
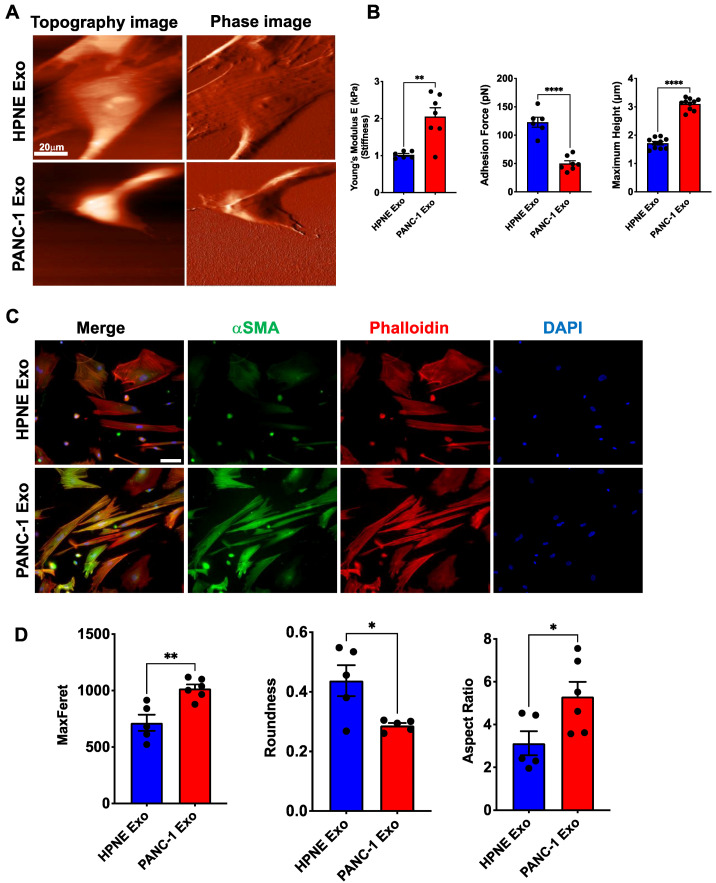
Biomechanical properties of pericytes are significantly influenced by cancer cell-derived exosomes. Force spectroscopy was performed on HPNE Exo- or PANC-1 Exo-treated pericytes (hPC) using AFM. (**A**) Representative AFM images showing the morphological changes in hPC on treatment with exosomes. Scale bar: 20 μm. (**B**) Young’s modulus *E* and the adhesion force were obtained from the force-distance measurements on the cell surface. Cross-sectional analysis of AFM images was used to measure the cell height after treatment with either HPNE Exo or PANC-1 Exo. (**C**) Representative images of exosome-treated hPC stained with Phalloidin and anti-αSMA Ab. Cells are counterstained with DAPI (blue) to visualize nuclei. Scale bar: 50 μm. (**D**) Morphological differences were measured by the max Feret, roundness and aspect ratio using ImageJ software. Unpaired two-tailed *t*-test was used to determine statistical significance and the data are represented as the mean ± SEM. * *p* < 0.05, ** *p* < 0.01, **** *p* < 0.000.

Our data show a significant increase in cellular stiffness on treatment with PANC-1 Exo (Figure 5B). Moreover, the cross-sectional analysis of AFM images along the central cytoplasmic region of the cell revealed that the cell body increases in height after exposure to PANC-1 Exo (Figure 5B). This result was consistent with stiffness changes in pericytes, reflecting that the alteration to the cell morphology was coupled with changes in the cytoskeleton structure induced by PANC-1 Exo treatment. The changes in cellular height and cytoskeleton structure are well represented in AFM images (Figure 5A). In addition, the detachment force required to separate the tip from the macromolecules on the cell surface during the FD curve measurements was used to determine the adhesion force of pericytes on exosome treatment. PANC-1 Exo-treated pericytes exhibited significantly less adhesion force (Figure 5B). To visualize cellular morphology changes and cytoskeletal arrangement, we immunostained exosome-treated pericytes for αSMA and phalloidin (Figure 5C). As we noted that PANC-1 Exo-treated pericytes were significantly stretched out, with prominent αSMA expression, we measured the cells’ maximum Feret diameter, roundness and aspect ratio (Figure 5D). Both the maximum Feret and aspect ratio were significantly increased when cells were treated with PANC-1 Exo compared to HPNE-Exo, whereas the roundness was decreased.

### 3.6. PDAC Cell-Derived Exosomes Are Sufficient to Induce αSMA Expression and Affect Vascular Integrity in Vivo

**Figure 6:**
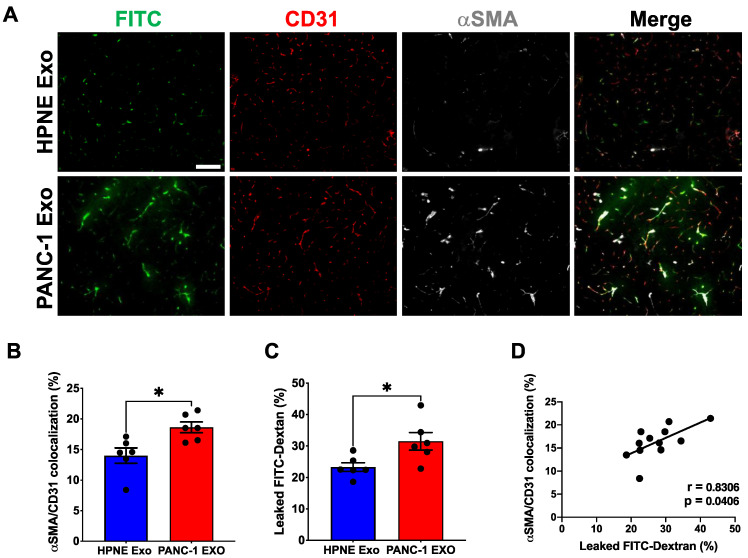
PDAC exosome is sufficient to induce pericyte αSMA expression and vascular leakiness in vivo. Immunodeficient NSG mice (non-tumor-bearing) were injected with either HPNE Exo or PANC-1 Exo every other day for four weeks, i.p. Mice were perfused with FITC-Dextran to measure permeability. Pancreatic tissues were stained for CD31 and αSMA. Leaked Dextran was visualized by FITC. (**A**) Representative images of pancreatic tissues from exosome-treated animals stained for CD31 and αSMA. (**B**) Quantification of percentage of CD31^+^ vessels associated with αSMA^+^ pericytes (HPNE Exo *n* = 6 mice; PANC-1 Exo *n* = 6 mice). (**C**) Quantification of percentage of FITC-Dextran leaked to the tissue (*n* = 6, all groups). (**D**) Correlation of FITC-Dextran leakage and αSMA^+^ pericyte-covered vessels. Pearson’s r correlation coefficient and significance levels are presented (*n* = 12). Unpaired two-tailed *t*-test was used to determine statistical significance and the data are represented as the mean ± SEM. Scale bar: 100 μm; * *p* < 0.05.

In vitro, PDAC cell-derived exosomes induced robust αSMA expression in the pericytes and affected the molecular, morphological and biomechanical properties. However, pericytes are part of blood vessels and have various functions together with endothelial cells, including mutual support. Thus, we sought to test if PDAC exosomes are sufficient to induce the αSMA expression in the pericytes of a normal pancreas in vivo. Exosomes from PANC-1 or HPNE cells were prepared and quantified by nanoparticle tracking analysis as described previously. To that end, 1 × 10^10^ exosomes suspended in 100 uL PBS were injected per mouse (WT NSG mice) every other day for four weeks, i.p. We did not note any histological changes in the pancreas or gross morphological changes in the blood vessels. However, αSMA expression in capillary-associated pericytes was significantly increased (Figure 6A,B). To investigate the functional consequence of αSMA-expressing pericytes on the vascular integrity, FITC-Dextran was injected right before collecting the pancreases. As indicated by the presence of the FITC signal within the tumor tissue outside of the vessels (Figure 6A,C), changes in the pericyte phenotype significantly increased the vascular leakiness in a normal pancreas, indicating that PDAC exosomes are sufficient to alter the pericyte phenotype in TME. The overall proportions of αSMA^+^ pericyte coverage and vessel leakiness were positively correlated (Figure 6D), confirming ectopic αSMA expression as a significant contributor to the pathological pericyte phenotype.

### 3.7. PDAC Exo-Treated αSMA^+^ Pericytes Exhibit Immunomodulatory Phenotype

**Figure 7:**
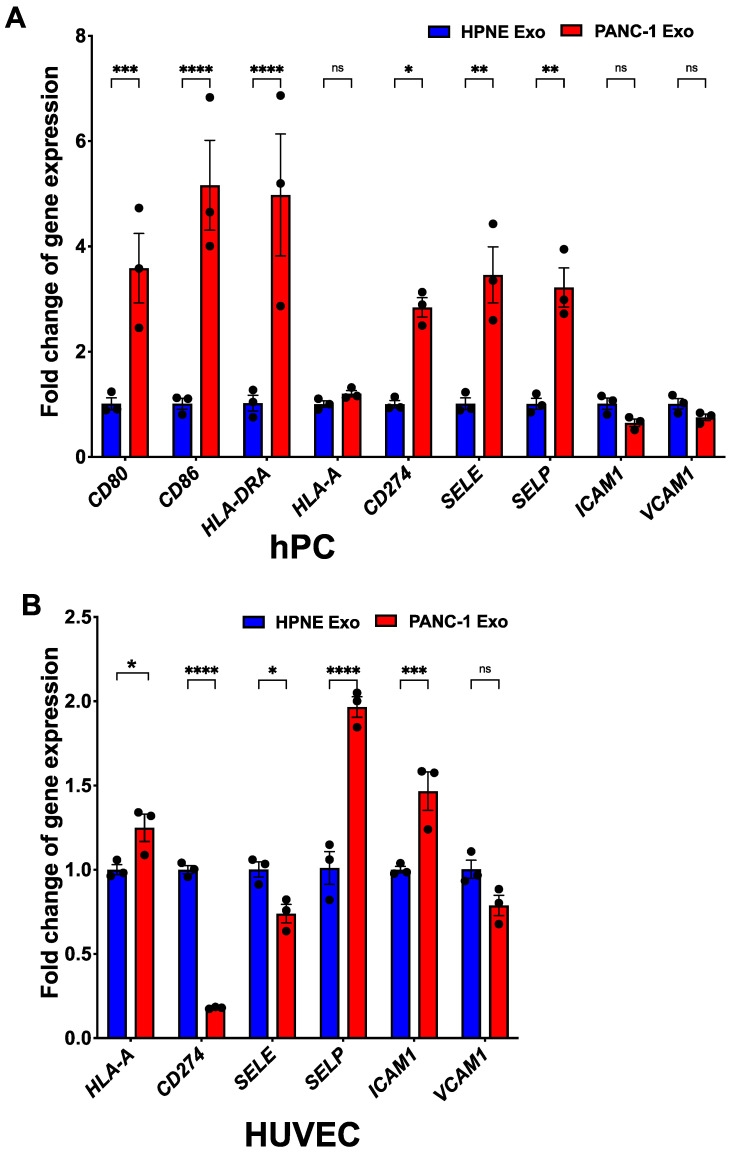
PDAC exosome-treated vascular cells acquired immune-related gene expression. Quantification of immunomodulatory and cell adhesion gene expression by (**A**) hPC or (**B**) hEC when treated with either HPNE Exo or PANC-1 Exo. Two-way ANOVA with Šídák’s multiple comparisons test was used to determine statistical significance and the data are represented as the mean ± SEM. * *p* < 0.05, ** *p* < 0.01, *** *p* < 0.00, **** *p* < 0.000, ns = not significant.

Immune cell trafficking is a highly selective process that is tightly regulated by endothelial cells and pericytes via adhesive interactions [41,42]. Aberrant pericyte phenotypes have been linked to the pathological function of blood vessels, including an immunosuppressive environment [53,54]. However, pericyte-mediated immunoregulatory effects have not been studied in PDAC. Thus, we also examined whether αSMA-expressing pericytes exhibit an immunomodulatory phenotype. Either HPNE Exo- or PANC-1 Exo-treated hPCs were analyzed for their immune-related gene expression by qRT-PCR. PANC-1 Exo-treated pericytes expressed significantly higher CD80, CD86, HLA-DRA and CD274, all immunoregulatory molecules. In addition, adhesive molecules such as E-selectin and P-selectin were significantly upregulated (Figure 7A). In the case of endothelial cells, P-selectin and ICAM-1 were significantly upregulated, representing an activated endothelial cell status (Figure 7B). Interestingly, CD274 was downregulated in PANC-1 Exo-treated endothelial cells. This result suggests that pathological pericytes represented by ectopic αSMA expression exhibit structural defects and altered immunoregulatory features.

## 4. Discussion

Accumulating evidence suggests that subpopulations of tumor pericytes undergo pathological phenotype switching induced by altered gene expression [24,34]. To gain insight into comprehensive perivascular phenotyping in PDAC, we examined the pericyte phenotypes of several mouse models and the human TMA of PDAC. Our study revealed that a significant portion of tumor vessels are covered by αSMA^+^ pericytes. We found the coverage was up to 73%, which is 13× higher than that of the normal pancreas (Figure 1 and Figure 2), implicating phenotype conversion during tumor progression. In addition, the αSMA^+^ pericyte coverage was significantly correlated with tumor hypoxia and vascular leakiness. While αSMA has been widely used as a pericyte marker, ectopic expression of αSMA in capillary pericytes [33] is known to confer a vascular smooth muscle cell (vSMC)-like phenotype [34,35]. A recent scRNA-seq study confirmed that normal brain pericytes do not express αSMA, and the absence of specific vSMC genes is the best way to define normal pericytes [32,55,56,57]. In brain pericytes, acquired αSMA expression correlates with conversion to a contractile arterial SMC (aSMC) phenotype, conferring disease-promoting properties [34]. These results strongly suggest that pericyte presence alone is not indicative of vascular maturity. In fact, pathological pericytes might do more harm than good for normal vascular function, which potentially leads to accelerated tumor progression and metastasis.

Despite the universal abnormalities of tumor-associated pericytes across tumor types, pericyte phenotypes vary in different tumors [24]. Pericytes are often identified by sets of molecular markers, but their expression pattern can be dynamic during development or disease progression [58]. In addition, different molecular subtypes of breast cancer, such as triple-negative breast cancer (TNBC) and luminal types, present strikingly different perivascular landscapes [23]. This evidence suggests that the perivascular landscape either results from an intrinsic property of the cancer cells or is influenced by the specific type of TME. Our effort to find the cause of the Desmin^low^ αSMA^high^ PDAC pericyte phenotype revealed that cancer cell-derived exosomes can induce significant αSMA expression in pericytes (Figure 4), suggesting the perivascular landscape is instructed by evolving cancer cells. Tumor-derived exosomes significantly influence distant organs to create a pre-metastatic niche [59], suggesting exosomes are a potent TME influencer. Most studies of cancer cell-derived exosome effects on stromal cells focus primarily on CAF and immune cells, and pericytes have yet to be explored in this context. 

In addition to the changes in the typical pericyte marker expression, PDAC-Exo-treated αSMA^+^ pericytes presented significant expression of immunomodulatory molecules, including PD-L1, HLA-DRA, CD86, CD80, E-selectin and P-selectin, which are absent from normal pericytes (Figure 7). Despite the presence of cytotoxic T cells [60], PDAC is overall immune-suppressive, partly due to the impaired activity of these immune cells and the presence of immune-suppressive cells [30]. Several studies showed that pericytes are intimately involved in immune cell recruitment, trafficking and activity [41,42]. B16 tumor-derived pericytes induce CD4^+^ T cell dysfunction or anergy [53]. Excessive VEGF expression in the TME affects the expression and function of adhesion molecules such as ICAM-1 and VCAM-1, hampering immune cell extravasation [61]. Studies on vascular normalization in mouse models of PNET showed that genetic deletion of Rgs-5 changed the intratumoral pericyte phenotype and enhanced the accumulation of CD8^+^ T cells after adoptive transfer [30]. 

While these results are encouraging and innovative, our data indicate that PDAC tumor-associated pericytes and PDAC Exo-treated pericytes do not express significant levels of Rgs-5, emphasizing pericyte heterogeneity across the different types of tumors. While αSMA expression itself cannot directly explain the acquired immunoregulatory gene expression, ectopic αSMA expression might represent an unexplored phenotype of pericytes in PDAC tumors that has acquired an immune-modulatory function. Such an indirect relationship can potentially be deciphered by using more comprehensive transcript screening such as single-cell RNA sequencing of PDAC tumor-associated pericytes. PDAC Exo also influenced the endothelial cells to a lesser degree, shown with a decrease in PECAM1 expression (Figure 4) and a significant increase in P-selection and ICAM1, both of which are indicative of activated endothelial cells. Combinatorial upregulation of CD274, P-selectin and E-selectin by pericytes might intercept extravasating T cells and negatively affect their activation and proliferation. The study of such a mechanism will require a complex co-culture system to examine T cell activity in the presence of tumor pericytes, along with high-resolution imaging technology to analyze the tumor vasculature in vivo.

αSMA is a cytoskeletal protein that can regulate cell stiffness, migration and downstream signaling such as Rec1-mediated signaling [15,62,63,64]. Our AFM data show a significant increase in pericyte stiffness and cellular height after exposure to PDAC Exo (Figure 5). PDAC Exo-treated pericytes also have an elongated morphology and cytoskeletal reorganization, as shown by phalloidin staining (Figure 5). Tumor-associated pericytes are loosely associated with the endothelial cells, with cytoplasmic processes that penetrate deep into the tumor parenchyma [16]. Perhaps acquired αSMA expression accompanied by morphological and biomechanical changes might contribute to such an aberrant anatomical position leading to non-functional or pathological tumor pericytes. Loose attachment of pathological pericytes will eventually lead to leaky vessels, which induce a hypoxic environment and increase the interstitial fluid pressure (IFP). Interestingly, a portion of the pancreatic islet capillary is associated with αSMA^+^ pericytes, which constrict capillaries and reduce the blood flow [35]. Blood vessels are constricted and collapsed within PDAC TME, which may be, at least in part, caused by an abundance of αSMA^+^ pericytes.

In summary, we have identified a unique perivascular phenotype in PDAC and revealed that the perivascular landscape can be shaped by cancer cell-derived exosomes, which makes perivascular landscaping one of the intrinsic properties of PDAC. As several studies have shown, anti-angiogenic approaches to deplete/prune tumor vessels without extreme caution will likely result in the progression of the disease. Instead, reverting a pathological phenotype to the normal phenotype might serve as a new vascular normalization approach with long-lasting efficacy when combined with chemotherapy or immunotherapy. To this end, identifying a unique molecular phenotype or target molecule of pathological pericytes will be an entry point to introducing a new therapeutic approach in the future.

## 5. Conclusions

PDAC TME presents an aberrant pericyte phenotype with high αSMA expression that can be induced by cancer cell-derived exosomes. A unique αSMA^+^ pericyte population presents altered biomechanical properties accompanied by an acquired immunomodulatory phenotype potentially contributing to hypoxic and immunosuppressive TME of PDAC.

## Data Availability

All data generated or analyzed during this study are presented within the article and its supplementary information files.

## Supplementary Materials

The following are available online at https://www.mdpi.com/article/10.3390/cancers14102448/s1, Figure S1. PDAC-associated pericytes exhibit aberrant molecular phenotype, Figure S2. Perivascular composition of PDAC tumor tissues, Figure S3. αSMA^+^ pericytes progressively appear on KPC tumors at different stages, Figure S4. αSMA^+^ pericytes’ coverage is correlated with hypoxia in PDAC tumors, Figure S5. Pericytes’ phenotype is influenced by pancreatic cancer cell-derived exosomes, Table S1. Primer sequences used for qRT-PCR.
