# Supplementary material for: Acquired αSMA Expression in Pericytes Coincides with Aberrant Vascular Structure and Function in Pancreatic Ductal Adenocarcinoma
Source: Cancers (Basel). 2022 May 16;14(10):2448. doi: 10.3390/cancers14102448 (PMC9139959; doi:10.3390/cancers14102448)
Supplement: Supplementary file 1 [file cancers-14-02448-s001.zip › cancers-1687358-supplementary.pdf]

SUPPLEMENTARY FIGURES AND TABLE

Figure 1S. PDAC associated pericytes exhibit aberrant molecular phenotype
Figure S2. Perivascular composition of PDAC tumor tissues
Figure S3. αSMA⁺ pericytes progressively appear on KPC tumors at different stages
Figure S4. αSMA⁺ pericytes coverage is correlated with hypoxia in PDAC tumors
Figure S5. Pericytes phenotype is influenced by pancreatic cancer cell-derived exosomes
Table S1. Primer sequences used for qRT-PCR

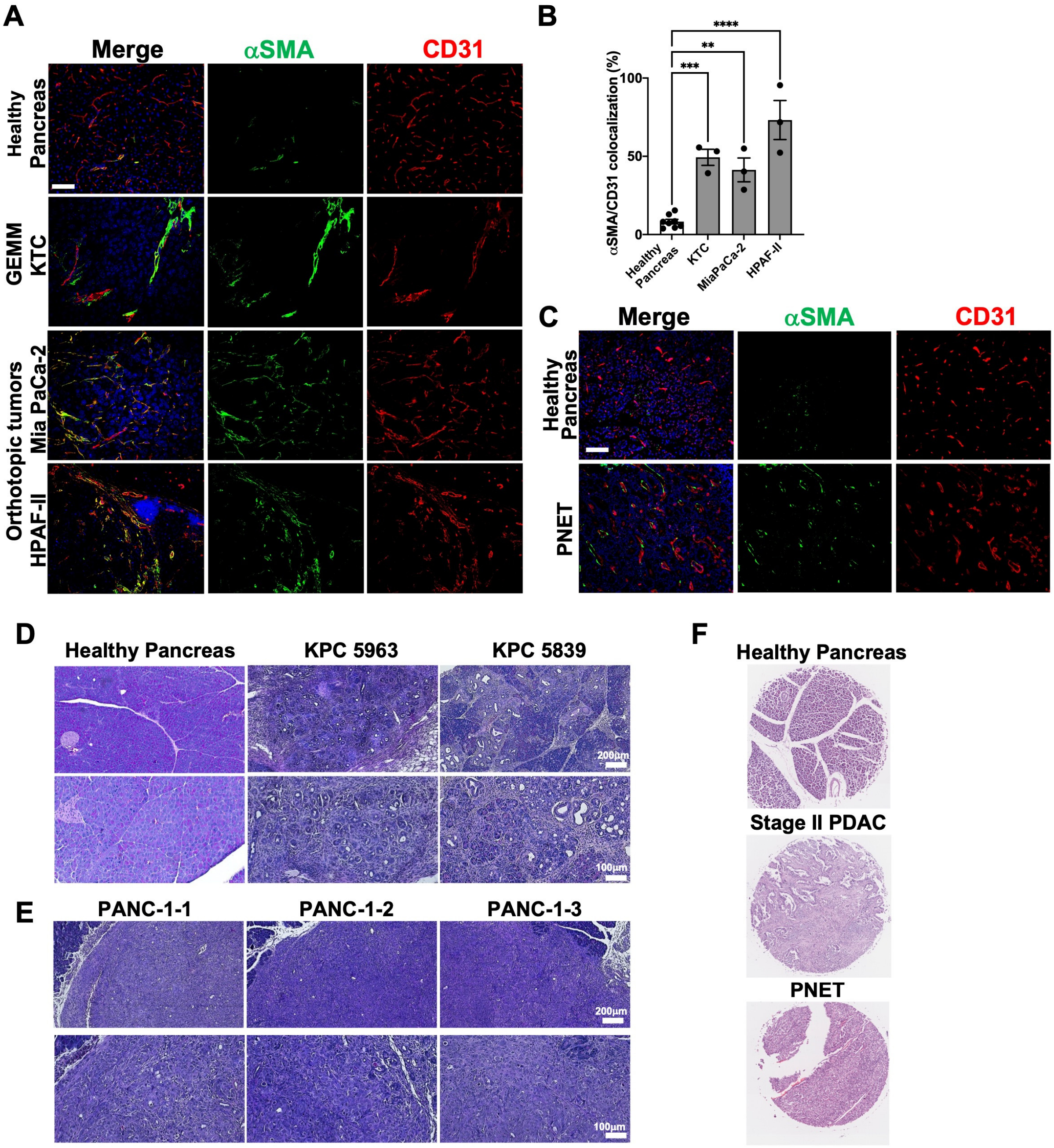

Figure S1. PDAC associated pericytes exhibit aberrant molecular phenotype. (A) Representative images of FFPE sections of murine PDAC tumors or healthy pancreas tissues immunolabeled for CD31 or αSMA. Scale bar: 100 µm. (B) Quantification of percentage of CD31⁺ vessels that are associated with αSMA⁺ pericytes (Healthy pancreas; n=8, KTC, Mia PaCa-2, and HPAF-II; n=3). The data are represented as mean ± SEM, and 1-way ANOVA with Dunnett's multiple comparisons was used to determine statistical significance. $^{**}P < 0.01$, $^{***}P < 0.001$, $^{****}P < 0.0001$. (C) Representative images of FFPE tissue microarray sections from normal human pancreas and pancreatic neuroendocrine tumors (pNET) immunolabeled for CD31 or αSMA. Scale bar: 100 µm. (D-F) Representative images of H&E staining of murine healthy pancreas tissues

and KPC tumor tissues (D), murine PANC-1 orthotopic tumor tissues (E), and normal human pancreas, PDAC, and PNET tumor tissues microarrays (F).

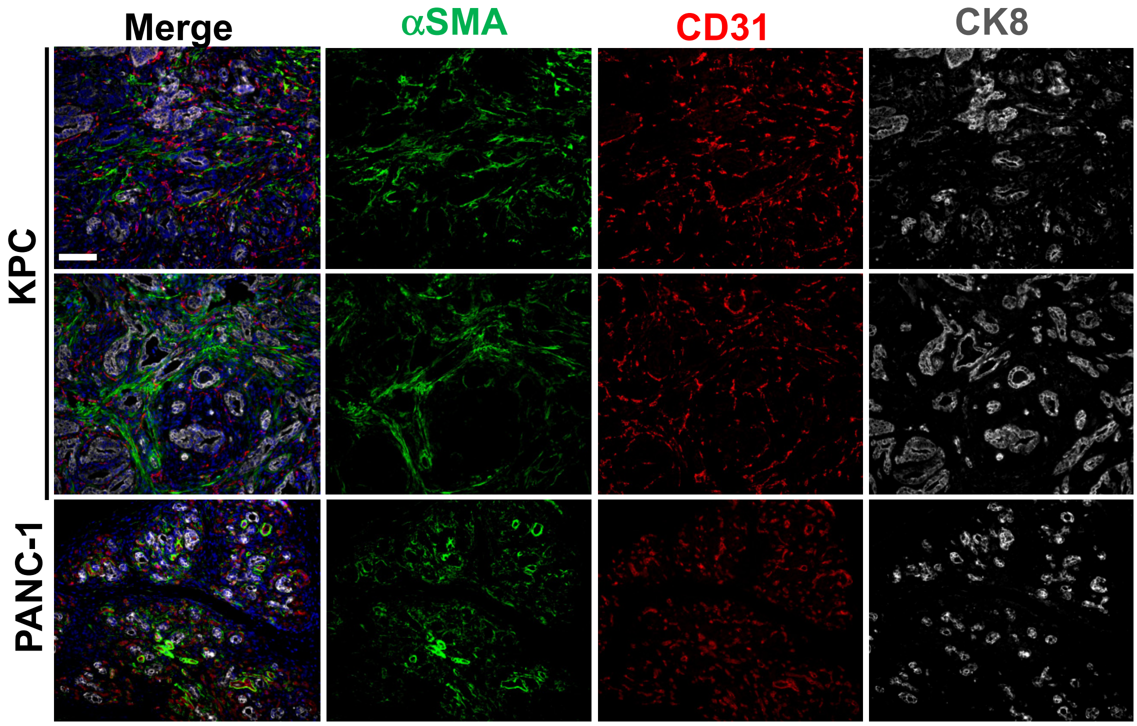

Figure S2. Perivascular composition of PDAC tumor tissues. Representative images of FFPE sections of KPC and PANC-1 orthotopic tumors immunolabeled for αSMA, CD31, and CK8 (cancer cells). Sections are counterstained with

DAPI (blue) to visualize nuclei. Scale bar: 100 μm.

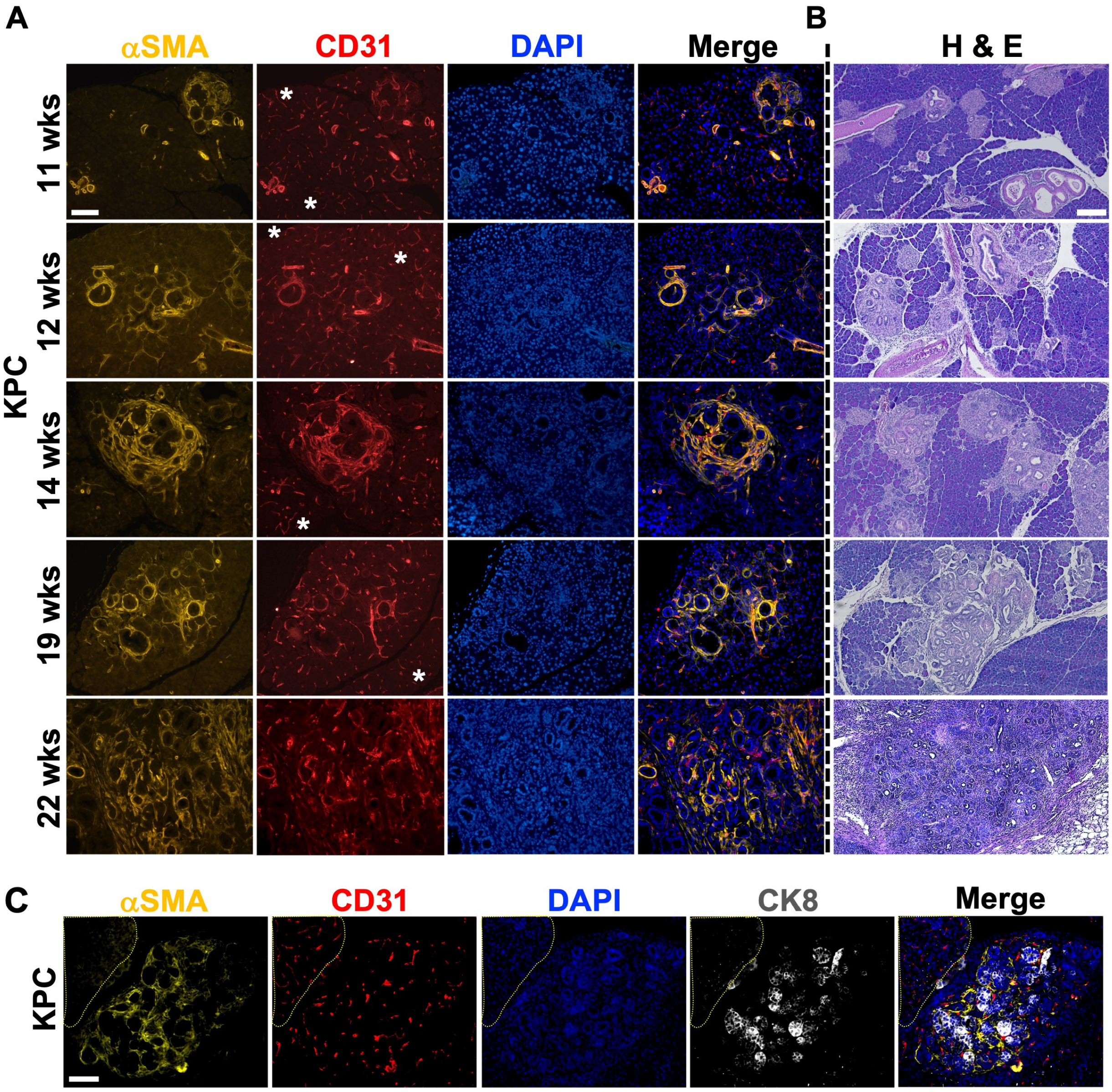

Figure S3. αSMA⁺ pericytes progressively appear on KPC tumors at different stages. (A) Representative images of FFPE sections of KPC tumors at different stages. Sections were immunolabeled for CD31 and αSMA. Sections are counterstained with DAPI (blue) to visualize nuclei. Asterisks indicate adjacent healthy pancreatic tissues with normal vessels without αSMA$^+$ pericytes coverage. Scale bar: 100 μm for all immunostained images. (B) Representative images of H&E staining of the KPC tumors at different stages. Scale bar: 200 μm. (C) Representative images of FFPE sections of KPC tumors immunolabeled for CD31, αSMA, and CK8 (cancer cells). Sections are counterstained with DAPI (blue) to visualize nuclei. The dotted yellow line delineates adjacent normal pancreas tissue (CK9 negative) containing vessels without αSMA$^+$ pericytes coverage. Scale bar: 100 μm.

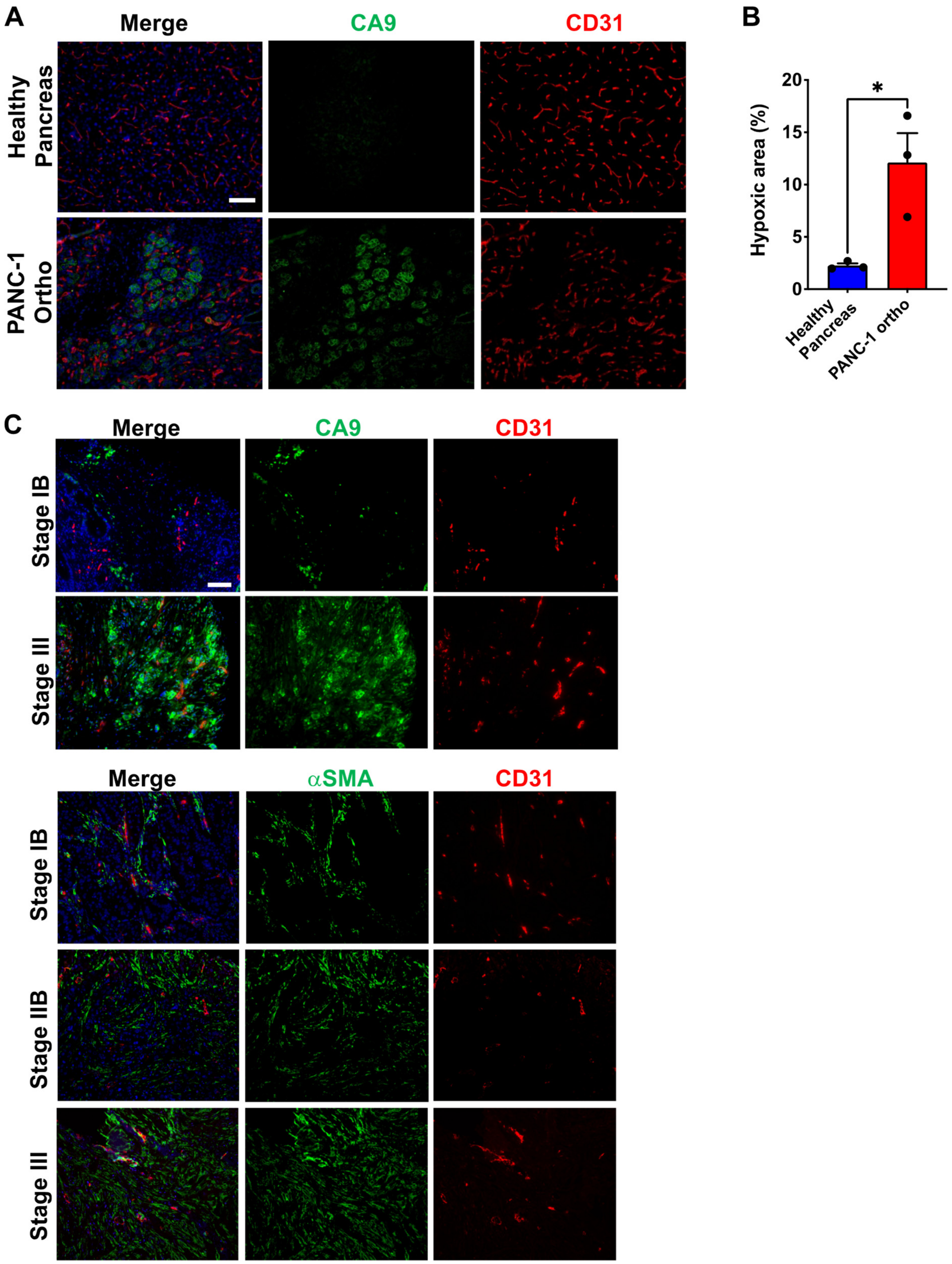

Figure S4. αSMA⁺ pericytes coverage is correlated with hypoxia in PDAC tumors. (A) Representative images of FFPE sections of PANC-1 orthotopic tumors and healthy pancreas. Sections were immunolabeled for CA9 and CD31. Scale bar: 100 µm (B) Quantification of percentage of hypoxic area ($CA9^+$ area per image) (n = 3, all groups). Unpaired 2-tailed t test was used to determine statistical significance. *P < 0.05 (C) Representative images of human PDAC tumors at different stages immunolabeled for CD31, αSMA, or CA9. Scale bar: 100 µm.

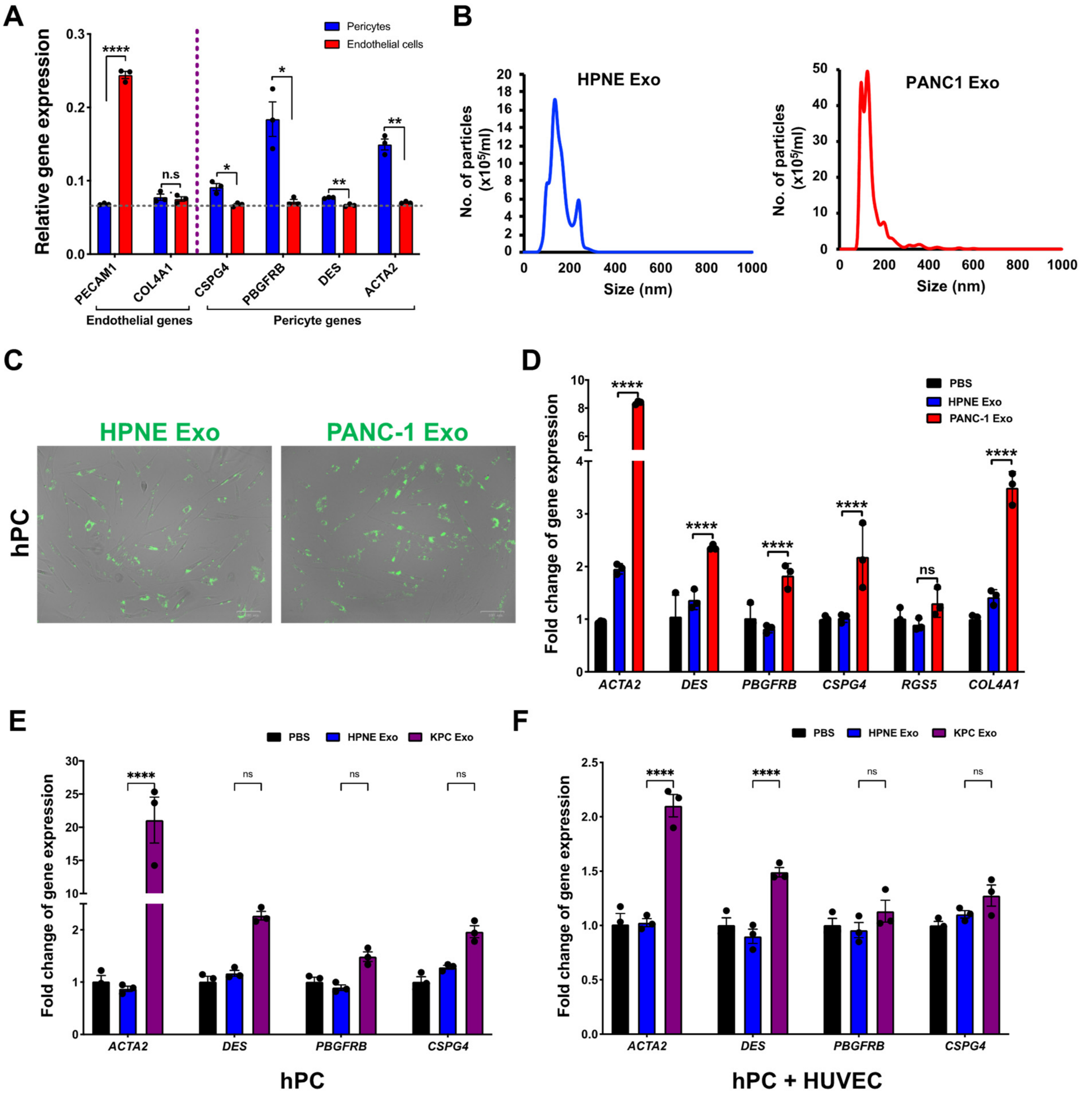

Figure S5. Pericytes phenotype is influenced by pancreatic cancer cell-derived exosomes. (A) Basal level gene expression of vascular cells. hPC and hEC were cultured in their optimal culture condition and gene expression was measured by qRT-PCR. Relative expression level was compared between hPC and hEC for each gene and statistical significance was determined by multiple unpaired t-test. (B) Exosome size and concentration distribution were measured using nanoparticle tracking analysis (NanoSight NS300). (C) Representative images of pericyte treated with DiO'; DiOC18(3) labeled exosomes. Bright field images to visualize the cells and green fluorescence images to visualize exosomes were superimposed. (D) hPC was treated with PBS, HPNE Exo, or PANC-1 Exo. Common pericytes marker expression was quantified by qRT-PCR. (E) Either hPC only or (F) hPC + hEC co-culture were treated with PBS, HPNE Exo, or KPC Exo. Quantification of pericyte marker gene expression in each condition. Unless otherwise stated, 2-way ANOVA with Tukey's multiple comparisons test was used to determine statistical significance and the data are represented as mean ± SEM. *P < 0.05, **P < 0.01, ***P < 0.00, ****P < 0.000, ns = not significant.

Table S1. Primer sequences used for qRT-PCR.

Gene		Sequences (5'-3')		Gene		Sequences (5'-3')
ACTA2	Forward	CTTCCCTGAACACCACCCAGTG		***VCAM1***	Forward	GGGAAGATGGTCGTGATCCTT
	Reverse	CATCGTCCCCAGCAAAGCCG			Reverse	TCTGGGGTGGTCTCGATTTTA
PBGFRB	Forward	AGCACCTTCGTTCTGACCTG		***SELE***	Forward	AGAGTGGAGCCTGGTCTTACA
	Reverse	TATTCTCCCGTGTCTAGCCCA			Reverse	CCTTTGCTGACAATAAGCACTGG
COL4A1	Forward	GGACTACCTGGAACAAAAGGG		***SELP***	Forward	ACTGCCAGAATCGCTACACAG
	Reverse	GCCAAGTATCTCACCTGGATCA			Reverse	CACCCATGTCCATGTCTTATTGT
CSPG4	Forward	CTTTGACCCTGACTATGTTGGC		***CD80***	Forward	AAACTCGCATCTACTGGCAAA
	Reverse	TGCAGGCGTCCAGAGTAGA			Reverse	GGTTCTTGTACTCGGGCCATA
DES	Forward	TCGGCTCTAAGGGCTCCTC		***CD86***	Forward	CTGCTCATCTATACACGGTTACC
	Reverse	CGTGGTCAGAAACTCCTGGTT			Reverse	GGAAACGTCGTACAGTTCTGTG
PECAM1	Forward	AACAGTGTTGACATGAAGAGCC		***HLA-DRA***	Forward	TTTCCGCAAGTTCCACTATCTCCC
	Reverse	TGTAAAACAGCACGTCATCCTT			Reverse	AATAATGATGCCCACCAGACCCAC
RGS5	Forward	CTTGCAGCTTTGCCCCACTC		***HLA-A***	Forward	CGACGCCGCGAGCCAGA
	Reverse	TCTTGGCTGGTTTCTCTGGCT			Reverse	GCGATGTAATCCTTGCCGTCGTAG
ANGPT2	Forward	ATCAGGACACACCACGAATG		***CD274***	Forward	TGGCATTTGCTGAACGCATTT
	Reverse	CATCCTCACGTCGCTGAATAA			Reverse	TGCAGCCAGGTCTAATTGTTTT
ICAM1	Forward	ATGCCCAGACATCTGTGTCC		***GAPDH***	Forward	GGTGTGAACCATGAGAAGTATGA
	Reverse	GGGGTCTCTATGCCCAACAA			Reverse	GAGTCCTTCCACGATACCAAAG
